# Individual stress response patterns: Preliminary findings and possible implications

**DOI:** 10.1371/journal.pone.0255889

**Published:** 2021-08-13

**Authors:** Rebecca Jacoby, Keren Greenfeld Barsky, Tal Porat, Stav Harel, Tsipi Hanalis Miller, Gil Goldzweig

**Affiliations:** Stress, Hope and Cope Lab., School of Behavioral Sciences, Tel-Aviv Yaffo Academic College, Tel-Aviv Yaffo, Israel; University of Cyprus, CYPRUS

## Abstract

**Background:**

Research on stress occupied a central position during the 20^th^ century. As it became evident that stress responses affect a wide range of negative outcomes, various stress management techniques were developed in attempt to reduce the damages. However, the existing interventions are applied for a range of different stress responses, sometimes unsuccessfully.

**Objective:**

The aim of this study was to examine whether there are specific clusters of stress responses representing interpersonal variation. In other words, do people have dominant clusters reflecting the different aspects of the known stress responses (physiological, emotional, behavioral, and cognitive)?

**Methods:**

The researchers derived a measure of stress responses based on previous scales and used it in two studies in order to examine the hypothesis that stress responses can be grouped into dominant patterns according to the type of response.

**Results:**

The results of Study 1 revealed four distinctive response categories: psychological (emotional and cognitive), physiological gastro, physiological muscular, and behavioral. The results of Study 2 revealed five distinctive response categories: emotional, cognitive, physiological gastro, physiological muscular, and behavioral.

**Conclusion:**

By taking into consideration each person’s stress response profile while planning stress management interventions and then offering them a tailored intervention that reduces the intensity of these responses, it might be possible to prevent further complications resulting in a disease (physical or mental).

## Introduction

The concept of stress in its various forms (such as anxiety and fear) has been known since the 18^th^ century but became central during the 20^th^ century. Stress models were, consequently, developed in order to explain the processes undertaken in response to stressors. Early stress research underlined the adaptive nature of organisms’ physiological response to acute stress and the potentially deleterious effects of prolonged stressors [1,2]. Later on, the research expanded to humans emphasizing the cognitive system as mediating between stressors and stress responses [3,4]. As it became evident that continuing or repeated stress responses affect a wide range of negative outcomes, both physical and psychological [5,6], various stress management techniques were developed in an attempt to reduce the harms [7–10]. These include psychophysiological techniques (such as relaxation, biofeedback and more) aiming to reduce stress responses and regaining control, as well as cognitive behavioral techniques aiming to challenge misleading perceptions. A substantial body of research has explored the efficacy of these techniques (e.g. [10]). However, in most cases, interventions were not tailored to specific patterns of responses, and thus, the same interventions were used, sometimes unsuccessfully, for various stress responses. Since stress responses influence organisms’ coping and health outcomes, their study is important. We believe that by better understanding the complexity of stress responses, appropriate interventions can be developed and implemented.

## Theories on stress responses

In the past, stress responses were studied and described mainly by physiologists,. Darwin (1809–1882), who studied the manifested responses of animals and humans to emotional situations, was mainly interested in the observable responses and not in the biochemical changes that occur in response to stress [11]. Immense strides toward understanding stress responses and their physiological basis were made by Cannon (1871–1945) and Selye (1907–1982). Cannon [2] studied the homeostatic mechanisms underlying the “fight or flight” response to stressful situations, while Selye [1] later developed the general adaptation syndrome (GAS) theory, which describes the process of responding to an ongoing stress.

According to these theories, exposure to a stressful event activates a series of autonomic system reactions that cause changes within organs [12,13]. The reactions found were the activation of the hypothalamic-pituitary-adrenal axis (HPA) and the sympathetic nervous system (SNS). Activation of the HPA axis and the SNS causes hormonal secretions of adrenaline and cortisol and the behavioral “fight or flight” reaction [2]. According to Berger et al. [14], the “fight or flight” response is triggered by osteocalcin, a protein released by the skeleton as a hormone, which, they claimed, is a messenger, sent by bone to regulate crucial processes all over the body, including how we respond to danger.

Porges [15] asserted that although these arousal theories have empirical support when measuring the effects of acute stress, they neglect other aspects of the physiological stress response such as parasympathetic nervous system (PNS) influences and interaction between sympathetic and parasympathetic processes. This neglect limits the theories’ ability to explain a wide variety of stress responses such as freezing, tonic immobility, fainting, and syncope. Porges’ polyvagal theory looks to explain the mechanism underlying the interpersonal differences of physiological and psychological stress responses. Other theories on the role of oxytocin for moderating the autonomic nervous system (e.g., [16]) and on gender differences, such as “tend and befriend” [17], have focused on responses directed toward safety behaviors.

Although these researchers have tried to include a psychological dimension in their models, this was mainly cast in terms of stimulus-response relationships, consistent with the dominant physiological and behavioral approaches of the period, and therefore could not explain why different people who are exposed to the same stimulus respond differently. These models, which were derived from animal behavior, were criticized for their universal approach of focusing solely on biological mechanisms and disregarding humans’ subjective perception of the stress experience [18].

From the middle of the 20^th^ century, the concept of stress came to occupy a central position in the psychological literature, and new stress models were developed emphasizing the interactions between individuals and their environment. The leading model in psychological stress research is the Transactional Model developed by Lazarus and Folkman [3]. This model focuses on the cognitive processes preceding the stress response and promotes the understanding of interpersonal variance in the stress responses to the same events. It emphasizes the importance of the individuals’ appraisal of the meaning of the stressful event and their own resources for coping with this event to help mediate between the stressor and stress responses of the organism. It also established the understanding that different individuals will react to the same event with varying intensity or duration: one will find an event threatening, while the other will find it neutral or realize that they have the required coping resources. The Transactional Model does not, however, explain the interpersonal variance of the stress response patterns and of stress influences on health.

Other theories have proposed a more integrative outlook on the stress-related cascade of events, starting even before the encounter with a potential stressor and resulting in various health outcomes. They have suggested a process that is mediated by cognitive appraisal, behavioral outcomes, and physiological mechanisms [19,20]. For example, Brosschot, Gerin, & Thayer [21] argue that perseverative cognition as manifested in worry, rumination and anticipatory stress should be considered as they are associated with enhanced cardiovascular, endocrinological, immunological, and neurovisceral activity. Others [22,23] have suggested that personality traits are also likely to influence how people respond to stress. These approaches consider all of the main aspects depicted by prior models and provide a wider perspective for both researchers and clinicians.

The aforementioned theories notwithstanding, individual differences of stress responses as represented by different clusters in a non-pathological population have not, to the best of our knowledge, been studied. The purpose of the current study was, therefore, to address this gap and examine whether reported stress responses do, in fact, reflect clusters of the common stress responses: physiological, emotional, behavioral, and cognitive. We also strived to assess interpersonal variation in stress responses; in other words, do people have dominant clusters of stress responses?

## Measuring stress responses

Different scales were developed to measure stress responses. For example, Terluin [24] developed the Four-Dimensional Symptom Questionnaire (4DSQ) in order to differentiate between general distress and what he considered as psychiatric symptoms, namely, depression, anxiety, and somatization. Schlebusch [25] developed the Stress Symptom Checklist (SSCL) which consists of three categories: physical, psychological, and behavioral. The checklist was intended to be a diagnostic tool that measures specific stress-related psychopathological conditions or disorders, particularly the intensity (or severity) of stress as reflected by an individual’s physical, psychological, and behavioral reactions.

These scales were primarily intended to measure the total intensity of the stress response in order to identify either pathological or intense stress responses, assuming the existence of a unified stress response for all. They ignored the different patterns people exhibit when confronted by a stressor, thus limiting their ability to characterize an individual’s dominant stress response pattern. Based on these works and others, stress responses were generally classified into four categories: physiological, emotional, behavioral, and cognitive [26,27].

## Methods

Two studies were conducted in order to examine the hypothesis that stress responses can be grouped into dominant patterns according to the type of response (physiological, emotional, behavioral, and cognitive). Although the existing scales include various items representing the above mentioned categories they are too long and didn’t meet our research purposes. Therefore we have decided to derive a short scale of stress responses, representing the four categories, based on the above mentioned scales (see details under " items selection" in the Study 1 description). Participants in the first study were students while participants in the second study were a sample of people suffering from the stress-related medical syndromes of fibromyalgia (FM), irritable bowel syndrome (IBS), or both. Participants in both studies were asked to rate the extent to which different stress responses characterize their typical responses to stress. The results are presented separately for each study.

The same statistical analysis was used for both studies. Descriptive statistics was calculated for each item (stress response). We conducted an exploratory factor analysis (principle component analyses with Varimax rotation) of all items. For the second study we also calculated sub-scale scores (base and factor analysis) and compared these scores between the study groups.

The two studies were approved by the ethics committee of the Academic College of Tel Aviv-Yaffo, and all participants signed an electronic consent form prior to the study’s initiation.

## Study 1

### Step 1: Items selection

In order to create a comprehensive list of stress response measures, the authors screened the two validated stress response questionnaires: the Four-Dimensional Symptom Questionnaire [24] and the Stress Symptom Checklist [25]. The responses were pre-classified separately by each of the authors into the four categories: physiological, emotional, behavioral, and cognitive. Discrepancies between the authors were discussed and resolved when at least four of the six authors agreed on the classification. Other items were newly added in order to encompass stress responses in all four categories. Four external experts examined and discussed the content validity of the new items as expressing stress responses and matching the relevant stress response categories. The final list included 66 items.

### Step 2: Identifying the partition of stress responses

#### Participants

The participants in Study 1 were first-year psychology undergraduate students at the Academic College of Tel Aviv-Yaffo. They participated in the study as part of their undergraduate program requirements and were recruited via the college’s credit database. A total of 100 participants enrolled in the study, with 91 fully completing the questionnaire. All 91 were first-year undergraduate students (84.6% female, 15.4% male) and the mean age was 23.56 years (SD = 1.37, range = 21–29).

#### Measures

The 66 selected items scale.

A short sociodemographic questionnaire including data on: age and gender.

#### Procedure

The items were presented to the participants through the Qualtrics^XM^ online platform. Participants were asked to recall a stressful event and to rate each response item on a scale ranging from “not at all” (1) to “always” (5) reflecting the extent to which each item characterize their response to stressful situations. All participants signed an electronic consent form before answering the questionnaires. The data was gathered and stored anonymously.

#### Data analysis

In the first stage of analysis we conducted an exploratory factor analysis (principle component analyses with Varimax rotation) of all 66 items, which revealed four factors (eigenvalue>1.0), accounting for 48% of overall variance. We then screened the items and excluded 36 items based on both content analysis and factor loadings (items with loading< 0.5 were omitted) following two-step analyses. We ended with a final set of 30 items which we found as satisfying for our research purposes (hereinafter the 30 items scale).

In the second stage of analysis, we determined the number of factors according to the Kaiser criterion of eigenvalue> = 1 [28] and identified 7 factors accounting for 70.38% of the total variance. However, according to the scree plot, 3–4 factors could have been retained. We chose a conservative approach and determined 4 factors. The 4 factors accounted for 58.48% of the total variance.

### Results

Table 1 presents the factor loadings and descriptive statistics for each item. It is evident that items loaded on Factor 1 include mostly psychological (emotional and cognitive) responses (introversion, loneliness, confusion, etc.). Factor 2 items include mostly physiological-gastro responses (digestive upset, stomach pains, etc.). Factor 3 items include mostly physiological-muscular responses (neck and shoulder pain, backaches, etc.). Factor 4 items include mostly unregulated behavioral responses (temper flare-ups, nervousness, etc.). The item “physical unrest” was loaded on both Factor 1 and Factor 2.

**Table 1:** Mean standard deviations and factor loadings of stress responses: Study 1.

	Mean ± SD	Factor 1	Factor 2	Factor 3	Factor 4
Tendency to introversion	2.75 ± 1.40	**0.80**	0.01	0.14	0.09
Loneliness	2.56 ± 1.48	**0.80**	-0.04	0.06	0.13
Decrease in enjoyment and/or desire to act	2.69 ± 1.26	**0.79**	0.21	0.04	0.08
Confusion	3.12 ± 1.29	**0.77**	-0.08	-0.06	0.20
Increase in negative thoughts	3.21 ± 1.30	**0.75**	0.28	0.03	0.18
Depression	2.84 ± 1.29	**0.75**	0.40	0.10	0.09
Emotional stress	3.66 ± 1.16	**0.69**	0.38	0.14	0.13
Difficulty concentrating	3.52 ± 1.12	**0.67**	0.11	0.16	0.38
Feeling of helplessness	3.04 ± 1.25	**0.66**	0.21	-0.14	0.17
Emotional flooding	3.71 ± 1.14	**0.65**	0.17	0.12	0.26
Anxiety	3.44 ± 1.36	**0.60**	0.36	0.29	0.12
Attention dispersion	3.32 ± 1.32	**0.57**	0.04	0.09	0.27
Crying spells	2.99 ± 1.32	**0.56**	0.19	-0.06	0.08
Insomnia	3.13 ± 1.34	**0.55**	0.35	-0.05	0.10
Fatigue, lack of energy	3.44 ± 1.19	**0.52**	0.40	0.13	0.20
Total Factor 1 (15 items)	3.16 ± 0.93				
Digestive upset (constipation/diarrhea)	2.47 ± 1.35	0.10	**0.80**	0.15	0.01
Stomach pain	2.52 ± 1.26	0.20	**0.76**	0.11	-0.16
Nausea	3.44 ± 1.19	0.21	**0.71**	0.25	0.11
Appetite change (over/under eating)	2.93 ± 1.29	0.23	**0.62**	0.25	0.24
Physical unrest	2.73 ± 1.36	**0.50**	**0.53**	0.21	0.15
Total Factor 2 (5 items)	2.56 ± 1.00				
Neck and shoulder pain	2.23 ± 1.34	-0.19	0.01	**0.78**	0.01
Backaches	2.13 ± 1.38	-0.08	0.08	**0.75**	0.04
Headaches	2.58 ± 1.26	0.28	0.03	**0.70**	0.01
Jaw tension	2.03 ± 1.25	0.21	0.26	**0.66**	0.03
Tight muscles	2.80 ± 1.31	0.06	0.40	**0.60**	-0.04
Teeth grinding	1.86 ± 1.12	0.08	0.21	**0.59**	0.09
Total Factor 3 (6 items)	2.27 ± 0.90				
Temper flare-ups	2.81 ± 1.18	0.25	0.02	-0.09	**0.83**
Irritability	3.31 ± 1.12	0.25	0.02	-0.15	**0.79**
Nervousness	3.59 ± 1.11	0.26	-0.03	0.24	**0.72**
Low frustration threshold	2.81 ± 1.22	0.19	0.18	0.16	**0.55**
Total Factor 4 (4 items)	3.13 ± 0.89				
Eigen value		7.76	3.68	3.33	2.78
% variance explained		25.86	12.26	11.11	9.25

We calculated a mean score for each factor. Factor 1, psychological, had the highest score (mean = 3.16; SD = 0.93; reliability Cronbach’s Alpha = 0.94, McDonalds Omega = 0.94), followed by Factor 4, behavioral, (mean = 3.13; SD = 0.89;reliability Cronbach’s Alpha = 0.84, McDonalds Omega = 0.84), and Factor 2, physiological-gastro, (mean = 2.56; SD = 1.00; reliability Cronbach’s Alpha = 0.65, McDonalds Omega = 0.67), and finally, Factor 3, physiological-muscular, (mean = 2.27; SD = 0.90; reliability: Cronbach’s Alpha = 0.77, McDonalds Omega = 0.79). Differences between all pairs of factors were significant except for Factor 3 vs. Factor 4.

In order to get further insight into the structure of the stress response items we conducted a smallest space analysis (SSA). SSA is a method of non-metric multidimensional scaling (NMDS) in which a set of variables and their inter-correlations are geometrically portrayed in a multidimensional space [29]. SSA treats each variable (i.e., each questionnaire item) as a point in a Euclidean space—the higher the correlation between two variables, the closer the points in the space. It attempts to find the space with the minimum number of dimensions in which the rank order of relations is preserved. The regional partition of the SSA space can be studied in conjunction with the corresponding content of the mapped variables. All points within a region should be associated with a specific set of variables of the same content [30–33].

**Fig 1:**
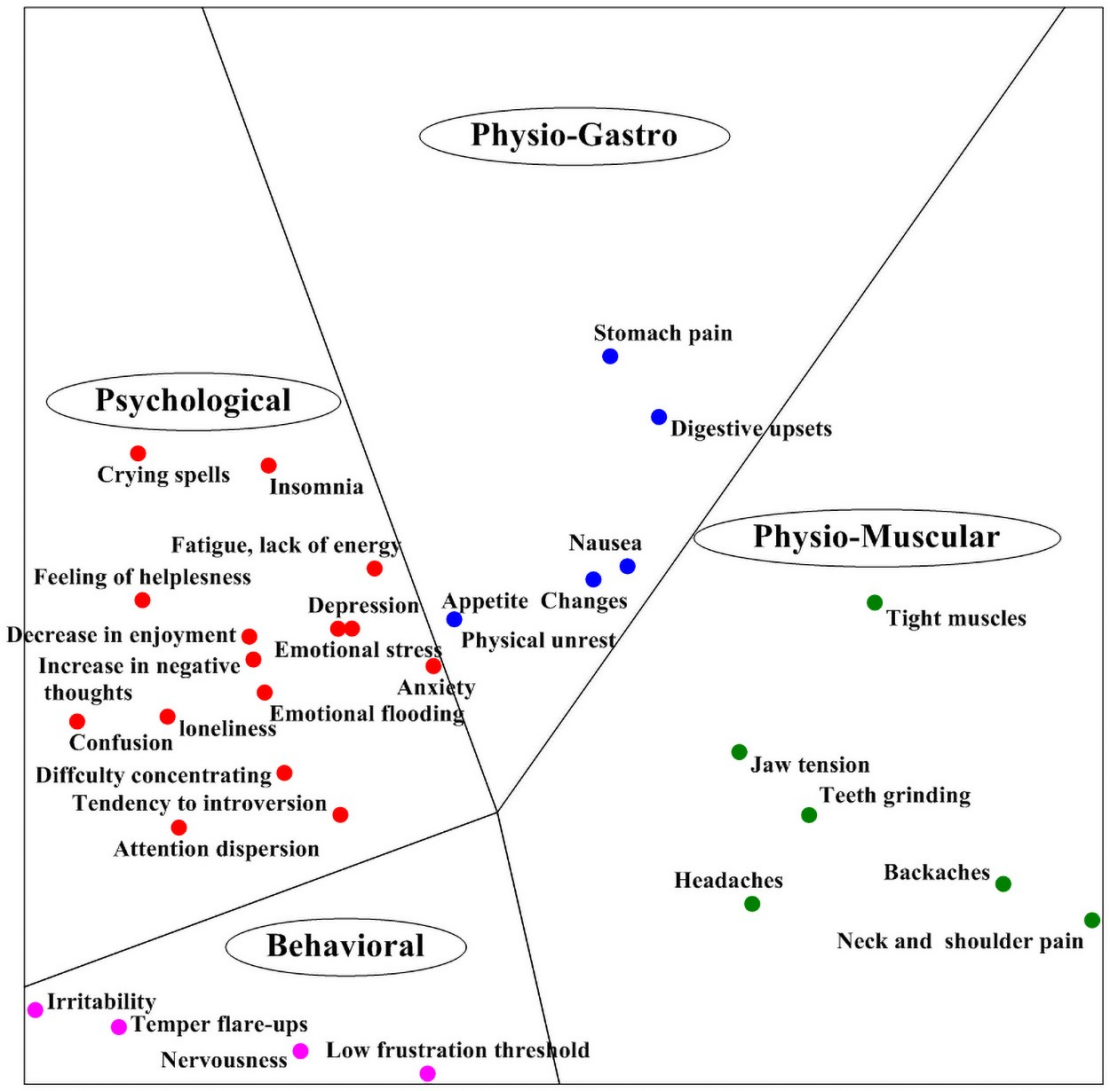
Map of smallest space analysis (SSA) of stress responses.

As can be seen, the SSA space in Fig 1 is partitioned into four polar (or angular) regions. Each polar region corresponds to one of the four categories—psychological, physiological-gastro, physiological-muscular, and behavioral—with their respective items. Polar regions divide the space into pie-shaped sections, all emanating from a common point. The elements of a polar facet are considered to be unordered but related [37]; they differ in kind but not necessarily in complexity. It should be noted that each two adjacent categories are close to each other in some respect.

## Study 2

### Participants

Participants were people over 18 years old diagnosed with fibromyalgia (FM), irritable bowel syndrome (IBS) or both. Participants were recruited from four different online forums based in Israel—two of which were dedicated to IBS the rest to FM. All participants volunteered for the study and none were offered any compensation. A total of 217 participants enrolled in the study but only 143 completed the questionnaire. Amongst these, 62 participants reported having FM (43.35%), 45 reported having IBS (31.47%) and 36 reported having both IBS and FM (25.17%). 95.1% of all participants reported being officially diagnosed by a doctor. The reported mean time from diagnosis was 8.43 years (SD = 6.82). 129 were female (90.2%) and 14 were male (9.8%). This gender difference might be partially explained by the fact that both IBS and FM are more common in women worldwide. Mean age was M = 37.67 years SD = 13.2.

### Measures

The 30 items scale (see Study 1).

A short sociodemographic questionnaire including data on: age, gender, diagnosis, time since diagnosis and who gave the diagnosis.

### Procedure

The items were presented to the participants through the Qualtrics^XM^ online platform. Participants were asked to recall a stressful event and to rate each response item on a scale ranging from “not at all” (1) to “always” (5) reflecting the extent to which each item characterize their response to stressful situations. All participants signed an electronic consent form before answering the questionnaires. All data was gathered and stored anonymously.

### Data analysis

We determined the number of factors according to the Kaiser criterion of eigenvalue> = 1 [36] and identified 7 factors accounting for 65.77% of the total variance. We identified 4–5 factors according to the scree plot. The fit to comparison data method (CD) revealed that the 4 factors solutions added significantly to the eigenvalue of 3 factors solution. Nevertheless, we decided on a conservative approach and we set the number of factors at 5. The 5 factors accounted for 58.01% of the total variance.

### Results

Table 2 presents the factor loadings and descriptive statistics for each item. Factor 1 included emotional responses identical to those included in factor 1 (psychological) in study 1. Three items that were included in this factor in study 1 (confusion, difficulty concentrating and attention dispersion) were now included in the additional factor 5 that consists of cognitive responses. Factor 2 included physiological- muscular items (identical to factor 3 in study 1) and the items insomnia and fatigue that were included in Factor 1 in study 1. Factor 3 included behavioural items and was identical to factor 4 in study 1. Factor 4 included physiological-gastro items, identical to the items included in Factor 2 in study 1 (except for the physical unrest item that in study 2 was included in Factor 1).

**Table 2:** Mean standard deviations and factor loadings of stress responses: Study 2.

	Mean ± SD	Factor 1	Factor 2	Factor 3	Factor 4	Factor 5
Increase in negative thoughts	3.34 ± 1.14	**0.75**	-0.02	0.11	0.01	0.23
Depression	3.15 ± 1.18	**0.74**	0.16	0.17	-0.09	0.10
Loneliness	3.18 ± 1.23	**0.72**	0.19	0.04	0.07	-0.13
Tendency to introversion	3.36 ± 1.09	**0.71**	0.08	-0.12	0.17	0.08
Emotional stress	3.95 ± 0.97	**0.67**	0.01	0.32	0.07	0.23
Feeling of helplessness	3.45 ± 1.07	**0.61**	0.19	0.20	0.24	0.09
Emotional flooding	3.70 ± 0.89	**0.56**	0.06	0.46	0.29	0.09
Crying spells	2.87 ± 1.13	**0.52**	0.13	0.36	0.18	-0.30
Decrease in enjoyment and/or desire to act	3.70 ± 0.97	**0.51**	0.15	0.14	0.30	0.37
Anxiety	3.56 ± 1.105	**0.50**	0.06	0.32	-0.06	0.28
Physical unrest	3.77 ± 1.04	**0.40**	0.30	0.01	0.26	0.31
Total Factor 1	3.46 ± 0.72					
Neck and shoulder pain	3.76 ± 1.34	0.02	**0.78**	0.06	-0.05	0.12
Backaches	3.43 ± 1.42	0.09	**0.76**	-0.04	-0.03	0.13
Tight muscles	3.90 ± 1.14	0.07	**0.68**	0.19	-0.08	0.15
Jaw tension	2.97 ± 1.46	0.15	**0.67**	0.03	0.18	0.13
Teeth grinding	2.34 ± 1.40	0.03	**0.63**	-0.12	0.30	0.06
Headaches	3.34 ± 1.17	0.00	**0.63**	0.10	0.15	0.11
Insomnia	2.08 ± 1.23	0.23	**0.56**	0.15	-0.11	0.02
Fatigue, lack of energy	4.28 ± 0.85	0.29	**0.53**	0.26	0.10	0.13
Total Factor 2	3.49 ± 0.85					
Irritability	3.36 ± 1.065	0.04	0.09	**0.83**	0.08	0.17
Temper flare-ups	3.05 ± 1.11	0.18	0.10	**0.80**	0.15	0.13
Nervousness	3.73 ± 0.90	0.17	0.14	**0.79**	0.01	0.14
Low frustration threshold	3.43 ± 1.05	0.38	0.05	**0.59**	0.05	0.27
Total Factor 3	3.395 ± 0.86					
Stomach pain	3.63 ± 1.21	0.08	-0.05	-0.04	**0.87**	0.07
Digestive upset (constipation/diarrhea)	3.76 ± 1.19	-0.01	-0.02	0.08	**0.81**	0.00
Nausea	2.92 ± 1.245	0.24	0.24	0.15	**0.57**	0.03
Appetite change (over/under eating)	3.63 ± 1.02	0.26	0.17	0.29	**0.53**	0.18
Total Factor 4	3.48 ± 0.88					
Difficulty concentrating	3.71 ± 1.025	0.10	0.21	0.23	0.16	**0.77**
Attention dispersion	3.50 ± 1.19	0.21	0.33	0.19	0.09	**0.73**
Confusion	3.04 ± 1.17	0.17	0.29	0.26	-0.02	**0.73**
Total Factor 5	3.42 ± 0.99					
Eigen value		4.86	4.098	3.38	2.625	2.49
% variance explained		16.02%	13.66%	11.27%	8.75%	8.30%

We calculated a mean score for each factor (see Table 2) there were no significant differences between the factors (largest difference was 0.0962). Factors reliability measures: Factor 1—Cronbach’s Alpha = 0.88, McDonalds Omega = 0.88; Factor 2—Cronbach’s Alpha = 0.82, McDonalds Omega = 0.83; Factor 3—Cronbach’s Alpha = 0.85, McDonalds Omega = 0.86; Factor 4—Cronbach’s Alpha = 0.74, McDonalds Omega = 0.75; Factor 5—Cronbach’s Alpha = 0.85, McDonalds Omega = 0.85.

In order to get further insight into the structure of the stress response items we conducted a smallest space analysis (SSA) similar to the SSA conducted in study 1.

**Fig 2:**
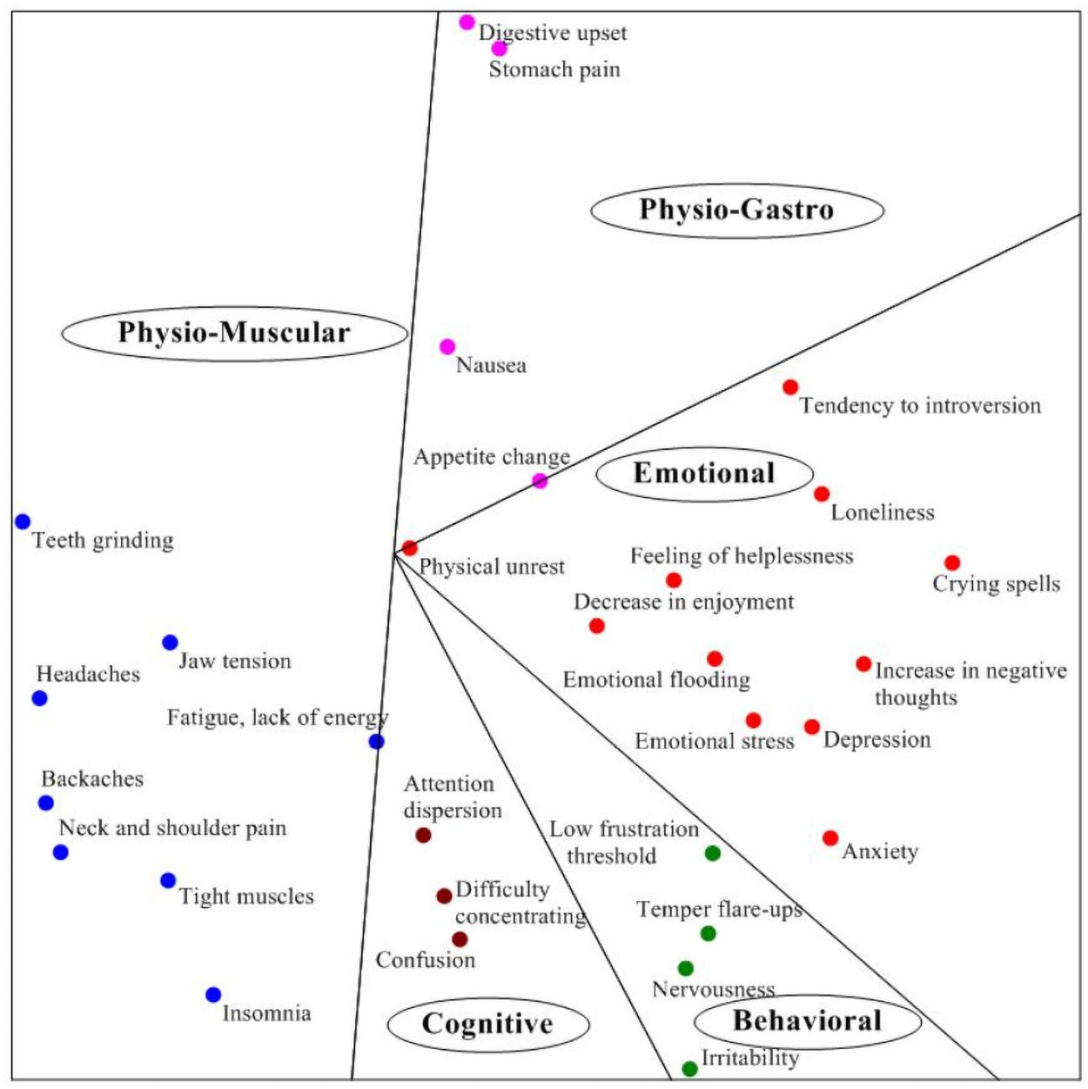
Map of smallest space analysis (SSA) of stress responses for study 2.

As can be seen, the SSA space in Fig 2 is partitioned into five polar (or angular) regions. Each polar region corresponds to one of the five categories—emotional, physiological-gastro, physiological-muscular, behavioral and cognitive—with their respective items. Polar regions divide the space into pie-shaped sections, all emanating from a common point. The elements of a polar facet are considered to be unordered but related; they differ in kind but not necessarily in complexity. It should be noted that each two adjacent categories are close to each other in some respect.

Table 3 which presents comparisons of the 5 factors between the study groups of study 2 indicate that the IBS group reported on significantly lower levels of physiological-muscular distress in comparison to the other two groups. The IBS group reported significantly higher levels of physiological-gastro distress in comparison to the FM group. The IBS group was also found to be significantly lower in comparison to the other two groups on the cognitive factor.

**Table 3:** Comparisons of stress responses between the study groups (study 2).

Stress response	IBS N = 45	FM N = 62	FM+ IBS N = 36	Univariate Analyses	Post Hoc Comparison* (Bonferroni)
	Mean ± SD	Mean ± SD	Mean ± SD	F(2,140) =; p<	
Emotional	3.32 ± 0.70	3.54 ± 0.70	3.50 ± 0.79	1.29; 0.28 N.S	IBS-FM p<0.36 N.S
					IBS-FM+IBS p<0.80 N.S
					FM-FM+IBS p<1.00 N.S
Physio- muscular	2.78 ± 0.82	3.78 ± 0.68	3.87 ± 0.60	32.98; 0.0001**	IBS-FM p<0.0001**
					IBS-FM+IBS p<0.0001*
					FM-FM+IBS p<1.00 N.S
Behavioral	3.20 ± 0.86	3.47 ± 0.83	3.51 ±0.90	1.74; 0.18 N.S	
Physio-gastro	3.76 ± 0.77	3.10 ± 0.90	3.80 ±0.71	12.115; 0.0001**	IBS-FM p<0.0001**
					IBS-FM+IBS p<1.00 N.S
					FM-FM+IBS p<1.00 N.S
Cognitive	2.93 ± 0.97	3.56 ± 0.89	3.78 ±0.99	9.37; 0.0001**	IBS-FM p<0.003**
					IBS-FM+IBS p<0.0001**
					FM-FM+IBS p<0.80 N.S

*P< 0.05;

**p<0.01;

G = Group; MANOVA overall stress responses was found significant: Wilks’ lambda = 0.45, F [5,136] = 13.315, P<0.0001.

Both the emotional and behavioral factors did not differ significantly between the groups.

## Discussion

In this study, we aimed to examine whether there are specific clusters of stress responses, representing interpersonal variation. More specifically, we hypothesized that different stress response clusters will reflect the different aspects of the known physiological, emotional, behavioral, and cognitive stress responses, thus allowing the identification of individual patterns.

The results obtained from Study 1 revealed four distinctive response categories: psychological (emotional and cognitive), physiological-gastro, physiological-muscular and behavioral. The psychological category entails mostly clinical symptoms of depression and anxiety as well as cognitive responses. Two of the categories are physiological responses: one mostly gastro-related symptoms and the other mostly muscle tension symptoms. The fourth category entails unregulated behaviors. The results obtained from Study 2 revealed five distinctive response categories: emotional, cognitive, physiological-gastro, physiological-muscular, and behavioral. As can be seen, the psychological category is divided into emotional and cognitive. These results thus portray an interesting classification that, if understood, may help to shed new light on stress response patterns and to highlight potential psychological and physiological susceptibilities.

It is already well established that psychological stress plays a role in negative physical and mental health conditions [20,34–36]. Thus, each of these response patterns may reflect a specific time point or dimension in the cascade of events vulnerability that may have a pathological outcome. For example, it was found that the link between stress and depression and anxiety is underlined by biological mechanisms such as HPA axis activation and inflammatory processes [37]. It can therefore be postulated that a person characterized by a high score in the emotional category may, in fact, be at risk of not only depression and anxiety disorder but also high cortisol-related illnesses. Such an assumption may be even more pronounced when an individual presents a high score in one or both of the physiological response categories. For instance, irritable bowel syndrome (IBS) was found to be adversely affected by psychological stress via several possible biological pathways including gastrointestinal function [38]. The digestion-related symptoms characterizing the physiological-gastro category may therefore, indicate susceptibility to such illnesses.

As a result of these findings, we decided to proceed with a study testing whether stress responses in a sample of people suffering from the stress-related medical syndromes of fibromyalgia (FM), irritable bowel syndrome (IBS) or both (FM+IBS) will reflect our assumptions.

Our results (see Table 2) indicate that both the IBS and the FM+IBS groups reported experiencing physiological-gastro stress responses during stressful events more often than the FM group. We also found that both the FM and the FM+IBS groups reported experiencing physiological-muscular stress responses during stressful events more often than the IBS group.

These results are compatible with previous research findings regarding pain sensitivity patterns in these groups. Two-thirds of IBS patients have been found to have lower visceral pain thresholds [39], while their musculoskeletal pain thresholds are normal [40] or higher than in normal controls [41]. In contrast, FM patients have decreased musculoskeletal pain thresholds but normal visceral pain thresholds [42,43]. It appears that only the subgroup of patients who have both IBS and FM suffer both from visceral and somatic hypersensitivity [42]. However, we find that the direction of the correlation needs further study. For example, it is possible that people who have physiological-gastro responses to stress are more likely to develop IBS later, but it is also possible that people who already have IBS are more likely to respond to stress with gastrointestinal symptoms. We suggest that future longitudinal studies inspect the nature of this correlation.

We also found that both the FM and the FM+IBS groups reported experiencing emotional stress during stressful events more often than the IBS group. An earlier finding by Janssens, Zijlema, Joustra, and Rosmalen [44] that major depressive disorder is more common in FM than in IBS may explain our findings.

## Conclusions

Our individual responses to stressful events embody much about who we are and what we have gone through. Our genetics, past experiences, gender, beliefs, and even smoking habits play a key role in how we react to stressors [19,45–47]. In mapping these reactions and patterns, we can obtain a clearer image of each person’s stress responses profile. A possible clinical implication of the findings of this study is the understanding that if we take into consideration the individual’s stress responses profile while planning stress management interventions and offer them a tailored intervention that reduces the intensity of these responses, we might prevent further complications resulting in physical or mental disease. Therefore, stress management interventions should be considered seriously and evidence based. An improved validated scale of stress responses may serve in the future as an important tool that will allow for the implementation of such tailored psychological interventions in various settings with minimal resources.

### Limitations

Despite these important implications, there are some methodological limitations in the current study. First, the scale we have used for our research purposes is composed of items selected from previous scales and has not been validated. Second, the participants in study 2 (people who suffer from FM or IBS or both) differ from those of study 1 (students). Third, the majority of the participants were female, which might have led to a bias due to gender differences.

In addition, there are some theoretical concerns. Since our results are based on retrospective reports, participants may not accurately remember how they usually act and feel and may appraise how they have always responded to stress according to the salience of events rather than actual frequency. Previous research has suggested that people who suffer from chronic pain have an attention bias that makes pain more salient to them than it would be in normal controls (e.g., [48]). We therefore propose that future studies use a daily log of stressful events and subsequent stress reactions in order to circumvent possible memory biases.

Another major challenge is differentiating between stress responses and stress coping strategies. Some theorists have even preferred to limit the concept of coping to voluntary responses [49], while others have included automatic and involuntary responses as well [50,51]. However, it is difficult to distinguish between voluntary and involuntary responses—if “volition” even exists at all. Libet [52] posited that if volition does indeed exist, it is only expressed when we use a conscious effort to think or behave differently than we are used to. Furthermore, thoughts and behaviors that are intentional and effortful when first used may, he claimed, become automatic and involuntary with repetition.

These limitations notwithstanding, our study calls for a detailed observation of the components of the existing stress models, focusing specifically on the function of stress responses and their impact on health while implementing tailored interventions that take into consideration individuals’ specific response clusters.
